# Assessment of construct validity and reliability of the Canadian Ultra-Processed Product Screener

**DOI:** 10.1017/S1368980026102304

**Published:** 2026-03-09

**Authors:** Virginie Hamel, Sara Desmarais, Sharon Kirkpatrick, Jane Y. Polsky, Carmen Byker Shanks, Maria Laura da Costa Louzada, Lana Vanderlee, David Hamond, Didier Gariguet, Jean-Claude Moubarac

**Affiliations:** 1 Départment de Nutrition, Université de Montréal, Montréal, Canada; 2 School of Public Health and Health Systems, University of Waterloo, Waterloo, Canada; 3 Department of Health Analysis Division, Statistics Canada, Ottawa, Canada; 4 Gretchen Swanson Center for Nutrition, Omaha, NE, USA; 5 Department of Nutrição, Universidade de São Paulo Faculdade de Saude Pública, São Paulo, Brazil; 6 École de Nutrition, Université Laval, Québec, Canada

**Keywords:** Ultra-processed foods, Construct validity, Reliability, Dietary assessment, Screener

## Abstract

**Objective:**

The Canadian Ultra-Processed Product Screener (CUPS) was developed to rapidly assess ultra-processed food (UPF) and drink product intake among Canadian adults. The CUPS is an online self-administered screener that includes twenty-eight questions and assesses the intake of a variety of UPF available in Canada, both in French and English. This study aimed to assess the construct validity and reliability of the CUPS among a sample of adults in Canada.

**Design:**

Cross-sectional study (between July and November 2023).

**Settings:**

Participants completed the online CUPS screener in three versions (1-d (twice), 7-d and 30-d CUPS) and three 24-h dietary recalls (24HR) (the reference measure) over the course of 26–28 d.

**Participants:**

354 Canadians aged 18–60 years

**Results:**

The CUPS had an acceptable construct validity, with moderate correlation coefficients between the CUPS score and UPF consumption level measured using multiple 24HR (from 0·33 to 0·44). Reproducibility was also acceptable (intraclass correlation = 0·61) and internal consistency ranged from good to excellent (Cronbach’s *α* = 0·72 for the 1-d and 0·86 for the 30-d CUPS). CUPS scores were also associated with higher intake of added sugars, saturated fats and Na.

**Conclusions:**

This study provides evidence supporting the construct validity and reliability of the CUPS among Canadian adults. The CUPS is useful for identifying low and high consumers of UPF and could serve as a proxy measure for one key dimension of diet quality, which is the type of food processing.

Ultra-processed food (UPF) and drink products are formulations of ingredients, mostly of exclusive industrial use, that result from a series of industrial processes^(1)^. Examples include carbonated drinks, fruit juices and drinks, candies, snacks, reconstituted meats, sauces and salad dressings^(1)^. High intake of UPF is associated with poor diet quality and increased risk of obesity and various non-communicable diseases, including diabetes, hypertension and certain types of cancers^(2)^. To evaluate UPF intake, the NOVA classification is used to classify food and drink products based on their level and purpose of processing^(3,4)^ and then the caloric contribution of UPF to total energy is quantified. However, dietary assessments using extensive questionnaires and 24-h dietary recalls (24HR) are generally time- and resource-consuming and, as such, infrequently conducted. For example, in Canada, the last nationally representative dietary intake survey was conducted in 2015. Dietary screeners, which are typically brief to implement, are known to reduce participant burden and research costs and tend to simplify analyses^(5,6)^. A brief screener measuring UPF intake specifically and rapidly among individuals and populations could facilitate research on UPF in Canada. Current food screeners in Canada such as the Canadian Food Intake Screener^(7,8)^ and the Canadian Eating Practices Screener^(8)^ tend to focus on key nutrients to limit and do not fully incorporate food processing into the survey items or analysis^(7)^. To address this gap, the Canadian Ultra-Processed Products Screener (CUPS) was developed to rapidly assess UPF intake among adults aged 18–60 years in Canada. The CUPS is an online self-administered screener and includes twenty-eight questions. Two versions were developed, examining intake according to different time frames: a simplified 1-d recall version and a frequency-based version assessing usual intake that can be applied to different time periods; both versions were available in English and French. Face and content validity assessment was conducted with a panel of fifteen experts, and cognitive testing with sixty potential users across Canada resulted in the final version of the CUPS (REF – Paper 1). Results from this study suggest that the CUPS is well understood in both languages (French and English), and that both versions (1-d and frequency-based) have similar face validity for users. The screener covers an extensive range of UPF that can be recognised by Canadians from different provinces with various levels of education (REF – Paper 1). The CUPS was developed to estimate UPF intake in settings where an assessment of the total diet (e.g. through a 24HR) is not feasible. It was also developed for use in settings that seek to assess levels of UPF consumption in cohort and intervention studies and evaluate the impact of food policies of Health Canada’s Healthy Eating Strategy (such as front-of-pack labelling) on UPF consumption^(9,10)^. The aim of this study was to assess the construct validity and reliability of the CUPS among a sample of Canadian adults.

## Methodology

### Design and overview

**Figure 1.**
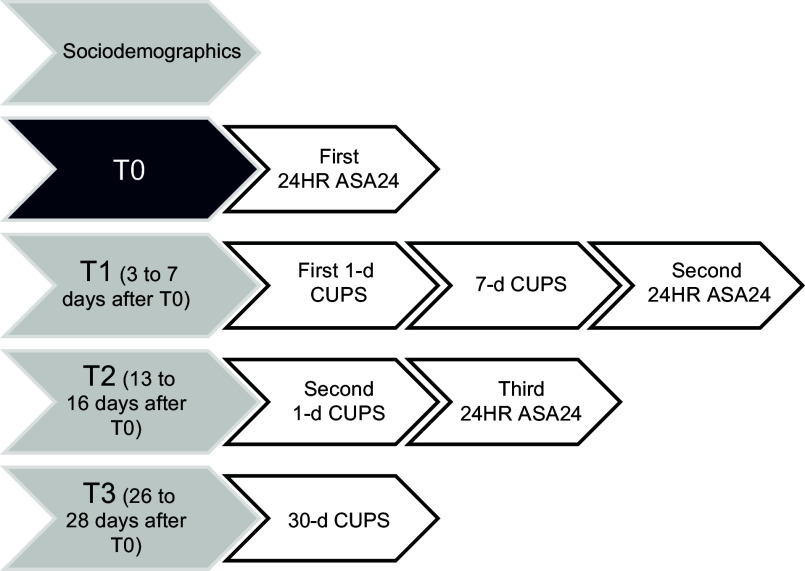
Summary of data collection steps and timeline. T, time; CUPS, Canadian Ultra-Processed Product Screener.

A cross-sectional study was conducted between July and November 2023 with a sample of Canadian adults. Participants completed eight questionnaires online in four steps during a month period (see Figure 1), including a sociodemographic questionnaire, four CUPS (1-d twice, 7-d and 30-d CUPS) and three 24HR using the Automated Self-Administered Dietary Assessment Tool (ASA24-Canada) adapted to the Canadian food supply^(11)^. The ASA24 is an adaptation of the Automated Multiple-Pass Method, which was developed for US national dietary surveillance and has been shown to support accurate recall of foods and beverages consumed^(12,13)^. Dietary data collected using a 24HR are known to be less affected by bias than other self-report methods (such as FFQ) while capturing the total diet^(14,15)^. For this study, the proportion of energy from UPF (% kcal from UPF on total kcal) was measured by the research team as described below, using ASA24, the reference measure of UPF intake, based on previous nutritional and epidemiological research on UPF^(1,3,16)^.

### Canadian Ultra-Processed Products Screener scoring system

The CUPS was tested in three versions: 1 d, 7 d and 30 d. The 1-d version asked about the consumption of twenty-eight UPF sub-categories on the previous day of the survey (‘Yesterday, did you have (…)?’) using ‘yes’ or ‘no’ response options. For each sub-category, a score of 1 was assigned for ‘yes’ and 0 for ‘no’, for a total score ranging from 0 to 28, with a higher score indicating higher intake of UPF. For the 7-d and 30-d versions, the questions were formulated as ‘Over the past week/month, how often did you have (…)?’ for the same twenty-eight UPF sub-categories. Table 1 presents the scoring systems of the three CUPS versions. The total score ranges from 0 to 112 for the 7-d CUPS and from 0 to 140 for the 30-d CUPS, with higher scores representing a higher frequency of UPF intake.

**Table 1.** The 1-d, 7-d and 30-d CUPS scoring system

	1-d CUPS	7-d CUPS	30-d CUPS
Question from the questionnaire	Yesterday, did you have any [UPF sub-category]?	Over the past week, how often did you have any [UPF sub-category]?	Over the past month, how often did you have any [UPF sub-category]?
UPF and drink products sub-categories	Fruit or vegetable juice drinks or iced tea Coffee or tea drinks Sports or energy drinks Soft drinks or flavoured sparkling water Chocolate milk Plant-based drinks Breakfast cereals Commercial baked goods Fast-food products Commercial breads Processed meat or seafood Plant-based meat and seafood alternatives Cheese products Instant, canned or packaged soups and noodles Frozen or fast-food pizzas Ready-to-cook or frozen meals Snack bars Flavoured yogurts or puddings Chips, crackers or other salty snacks Chocolates or candies Ice cream, popsicles or frozen desserts Spreads or jams Margarine or shortening Sauces or condiments Salad dressings Broths or seasonings Sugar substitutes or coffee whiteners Protein powder or meal replacements		
Potential responses [associated score]	[0] – No [1] – Yes	[0] – Never [1] – 1–2 times [2] – 3–4 times [3] – 5–6 times [4] – Once a day or more	[0] – Never [1] – 1–3 times over the past month [2] – 1–2 times a week [3] – 3–4 times a week [4] – 5–6 times a week [5] – Once a day or more
Score range	0–28 points	0–112 points	0–140 points

CUPS, Canadian Ultra-Processed Product Screener; UPF, ultra-processed food.

### Sample size calculation

To determine the sample size, the effect size of our main analysis (correlation between the CUPS and our reference measure, i.e. % kcal from UPF) was estimated to be of a medium order (0·5), based on studies reporting a medium/strong negative correlation (–0·47 and –0·74) between similar diet quality indices and proportion of calories from UPF^(17,18)^. Based on a hypothesised correlation of at least 0·4 (null hypothesis of 0·2, with an *α* of 0·05 and power of 90 %), a correlation of 0·375 can be detected (or an effect size of 0·355). This number was inflated by a factor of 2 to account for validity assessment in both languages and a further 20 % for poor quality data and attrition following recommendations^(19)^, resulting in a total sample of 398.

### Sampling and data collection

Inclusion criteria were (a) being an adult aged between 18 and 60 years and (b) living in Canada for at least 1 year to avoid bias caused by unfamiliarity with food products available in Canada. Children, adolescents and older adults were excluded due to different cognitive demands and memory biases that would require specific validation^(20)^. Participants were recruited through Léger Opinion^(21)^ in four consecutive rounds between July and November 2023 using quotas for sociodemographic characteristics, including age, sex, province of residence, level of educational attainment and language. Quota sampling was employed to recruit an equivalent proportion of males and females, along with a balanced representation of age groups and provinces representing the Canadian population’s sociodemographic characteristics^(22,23)^. Individuals from the territories were not included because the CUPS was based on national data surveys that did not include these territories^(24)^. Quota sampling was also used to select about 20–25 % of the sample who completed the study in French (to ensure our sample did not over-represent individuals from Quebec, the main French-speaking province) and about 20–25 % whose highest level of education was high school. Sociodemographic characteristics were collected through Léger Opinion’s eligibility questionnaire. Eligible and interested participants were sent information about the study and deadlines for completing each questionnaire by email.

The CUPS were hosted on a platform developed by Polygon, a company that specialises in data collection and analysis tools and complies to research requirements (i.e. anonymity, user functionality and datasets)^(25)^. At T0 (Figure 1), participants received a link to complete the first 24HR through the ASA24-Canada platform. Three to seven days later, at T1, participants were asked to complete the 1-d and 7-d CUPS on Polygon and a second 24HR through the ASA24-Canada platform. At T2, 13–16 d after T0, they were prompted to complete the 1-d CUPS and a third 24HR on ASA24. Finally, at T3, 26–28 d after the first step, participants completed the 30-d CUPS. Participants were compensated by Léger Opinion with 50 000 LEO points (equivalent to 50 Canadian dollars) for completing the four steps.

**Figure 2.**
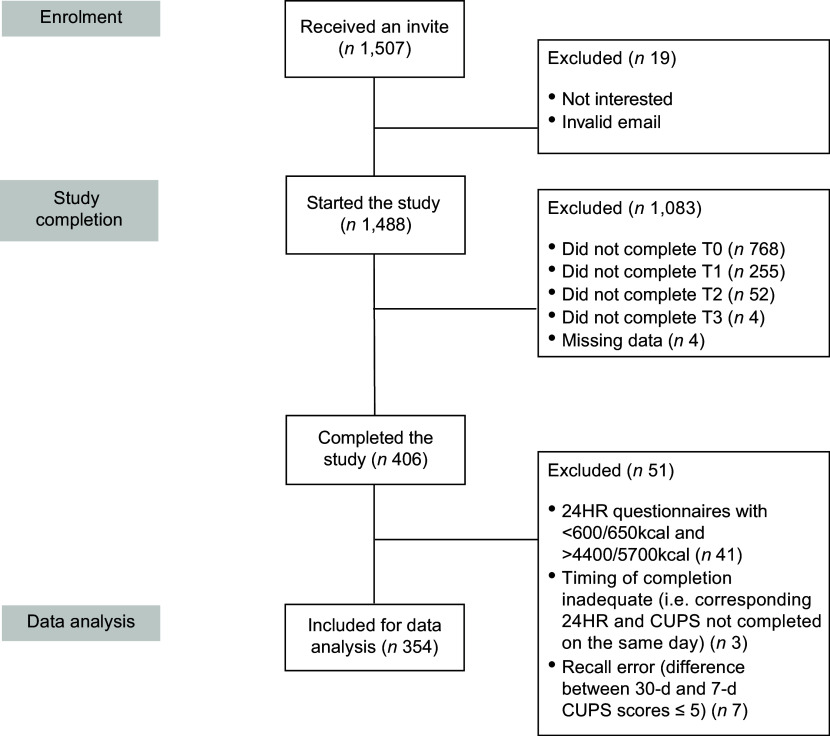
Flow chart describing the participation in the study. CUPS, Canadian Ultra-Processed Product Screener.

A reminder email was sent 2 d after the first mailing and a second 4 d later. Emails were also sent if participants partially completed a step (such as completing one out of three questionnaires at T1 by the deadline). Participants received a username and a password for each questionnaire, which allowed us to match their data from the CUPS and ASA24 platforms. This also enabled the anonymisation of participant data for analysis. A total of 1507 potential participants completed the eligibility questionnaire, and 406 completed all four study steps (Figure 2). Participants were excluded if they did not complete all required questionnaires within the designated time at each step (*n* 1083). Following the National Cancer Institute guidance for reviewing and cleaning ASA24 data, forty-one participants with estimated energy intake below 600 and 650 kcal a day or above 4400 and 5700 kcal for women and men, respectively, were excluded^(26)^. Data for three participants were excluded due to a mismatch between 24HR and CUPS completion times. A small number of participants were excluded (*n* 7) due to inconsistent data at T3, indicative of fatigue (i.e. when the difference between the 30-d and the 7-d CUPS scores was negative; for example, a score of 5 at the 7-d CUPS but zero at the 30-d CUPS). Data from participants who started but did not complete all time points were excluded for most analyses, except for internal consistency analyses, where all completed 1-d, 7-d and 30-d CUPS data were used (Table 2).

**Table 2.** Internal consistency of the 1-d, 7-d and 30-d CUPS scores

CUPS version	Cronbach *α*	*n*	*F*	*P* value
1-d CUPS at T1	0·72	565	42·4	< 0·001
1-d CUPS at T2	0·63	441	34·3	< 0·001
7-d CUPS	0·80	536	63·9	< 0·001
30-d CUPS	0·86	410	70·2	< 0·001

CUPS, Canadian Ultra-Processed Product Screener.

### Food classification of the ASA24 data according to the NOVA classification

All foods and drinks (hereafter referred to as ‘foods’) reported in the three 24HR using ASA24 were classified according to the NOVA classification^(27)^, which groups foods according to the extent and purpose of industrial processing^(3)^. Foods were classified into four distinct groups: (1) unprocessed and minimally processed foods, such as frozen fruits and vegetables, plain milk, pasta and flour; (2) processed culinary ingredients, including oils, butter, sugar and salt; (3) processed foods, such as canned vegetables, canned fish, fruits in syrup, cheese and freshly made artisanal bread; and (4) UPF, including mass-produced industrial breads and buns, reconstituted meat products, commercial fruit juices and fruit drinks and confectionary (e.g. chocolate, candies and desserts). Further details on the NOVA classification methods have been previously published^(4,28)^.

### Statistical analyses

Statistical analyses were conducted using SPSS, version 29.0.1.0^(29)^. Descriptive analyses were generated to assess participants’ characteristics, CUPS mean scores and completion time. Construct validity and reliability assessment analysis are described below.

#### Construct validity assessment

Construct validity was assessed to determine whether the CUPS performs as expected based on the theory underlying its development, that is, can the tool accurately estimate the consumption level of UPF and distinguish between low and high consumers^(20)^. Three analyses were conducted for this purpose. First, the performance of the three versions of the CUPS in estimating UPF intake was compared against the reference UPF intake method (% kcal from UPF from 24HR). Spearman’s correlations were used to assess the correlation between scores obtained from the CUPS and the corresponding 24HR from ASA24 (completed at the same time (T), see Figure 1). The 1-d CUPS at T1 was matched with the 24HR at T1, the 1-d CUPS at T2 was matched with the 24HR at T2, the 7-d CUPS was compared to the two 24HR at T0 and T1 and the 30-d CUPS was compared to the three 24HR at T0, T1 and T2. We also evaluated the correlation coefficients between the mean scores of the 1-d CUPS at T1 and T2 and the mean % of kcal estimated from the corresponding 24HR at T1 and T2. We conducted analyses for the whole sample and stratified analyses by sex, age, educational attainment, language and province to assess if the scores could detect differences across sociodemographic groups using the screeners and the 24HR. For this, ANOVA tests were used to compare the mean CUPS scores and mean % kcal calculated from the 24HR across these groups (Table 3). Where evidence indicated a difference, a Bonferroni *post hoc* test was conducted. Pearson’s correlations, stratified by sociodemographic groups, were used to assess the correlation between the CUPS and the reference measure scores to examine its performance across sociodemographic groups.

**Table 3.** Mean proportion of daily energy from ultra-processed food and (UPF) drink products and mean 30-d CUPS scores at T0, T1 and T2 according to sociodemographic characteristics (*n* 354)

			Energy contribution from UPF (% kcal/d), Mean (sd)									CUPS score, Mean (sd)								
	Distribution of participants % (*n*)		24HR at T0		24HR at T1		24HR at T2		Mean for T0,T1 and T2 based on 24HR		*P* value§	1-d CUPS at T1‡		1-d CUPS at T2‡		7-d CUPS‡		30-d CUPS‡		*P* value^||^
	%	*n*	Mean	sd	Mean	sd	Mean	sd	Mean	sd		Mean	sd	Mean	sd	Mean	sd	Mean	sd	
Sex at birth																				
Male	43·5	154	46·0	23·8	50·0	24·0	50·0	23·5	49·2	17·8	0·669	5·4	3·3	5·4	3·1	18·7	9·1	29·5	14·8	0·285
Female	56·5	200	47·5	22·9	47·8	23·5	50·2	22·5	48·4	16·3		5·5	3·6	4·7	2·7	17·9	8·5	27·9	12·9	
Age (years)																				
18–29	21·8	77	43·9	23·2	45·6	25·7	48·7	23·2	46·4	19·8	0·425	5·3	4·4	4·4	2·6	17·5	7·9	26·6	12·1	0·221
30–39	22·0	78	48·5	26·1	52·7	25·3	49·6	24·6	50·2	16·9		5·7	2·9	5·7	3·5	19·1	8·7	31·14	14·2	
40–49	28·8	102	49·2	23·3	48·1	22·8	51·1	23·3	50·0	15·7		5·5	3·3	5·0	3·2	18·2	8·5	28·3	13·1	
50–59	27·4	97	45·4	20·7	48·8	21·5	50·6	20·9	48·0	15·6		5·4	3·3	4·9	3·0	18·1	9·8	28·5	15·0	
Educational attainment																				
High school or less	22·6	80	46·9	22·0	50·6	26·4	53·1	24·4	50·2	17·9	0·182	5·4	3·8	4·9	3·1	18·6	10·3	28·8	14·9	0·741
Some post-secondary*	26·8	95	52·3	21·8	49·1	21·6	50·2	22·6	50·6	15·9		5·6	3·6	4·7	3·0	18·5	9·1	29·4	14·7	
University degree or above	50·6	179	43·9	24·2	47·8	23·6	48·7	22·3	47·1	16·9		5·5	3·3	5·2	3·2	17·9	7·8	28·1	12·6	
Language																				
English	77·4	274	46·8	23·2	48·4	24·3	50·6	23·2	48·7	17·0	0·960	5·3	2·6	4·9	3·5	18·2	8·0	28·9	13·7	0·501
French	22·6	80	46·9	23·7	50·0	21·6	48·5	21·6	48·8	16·8		5·5	3·7	5·1	3·0	18·2	9·0	27·7	13·7	
Province†																				
Atlantic provinces	11·3	40	52·7	23·0	56·4	23·8	56·2	21·1	54·8	15·6	0·006	5·6	2·7	5·4	2·9	19·1	8·1	33·6	13·5	0·070
Quebec	20·3	72	47·1	22·6	47·4	21·9	47·3	21·7	47·7	17·3		5·1	2·4	4·8	3·3	18·0	7·9	27·3	13·0	
Ontario	20·9	74	47·1	22·4	47·7	25·1	54·4	23·8	50·1	18·3		5·5	4·3	5·0	3·2	18·8	10·4	29·6	15·1	
Prairie provinces	28·2	100	48·2	25·2	49·3	24·8	52·3	23·0	50·0	16·8		5·9	3·7	5·1	2·7	18·9	8·2	28·8	12·1	
British Columbia	19·2	68	41·0	21·5	46·2	22·1	41·5	21·7	42·9	14·3		5·1	3·6	4·9	3·5	16·4	9·0	25·8	14·8	
Total sample	100	354							48·7									28·6	13·7	

CUPS, Canadian Ultra-Processed Product Screener.

* Post-secondary includes trade certificate or diploma, college, CEGEP (College of general and professional education unique to Quebec province) or other non-university certificate or diploma, and university certificate or diploma below bachelor level.

† Atlantic provinces include New Brunswick, Prince Edward Island, Nova Scotia, and Newfoundland and Labrador. Prairie provinces include Alberta, Saskatchewan and Manitoba.

‡ *P* value for ANOVA test for differences in the mean % kcal from UPF across sociodemographic subgroups.

§ The score ranged from 0 to 28 for the 1-d CUPS, 0 to 112 for the 7-d CUPS and 0 to 140 for the 30-d CUPS.

|| *P* value for ANOVA test for differences in the mean 30-d CUPS scores across sociodemographic subgroups.

Whether the CUPS scores captured enough variation to distinguish between low and high consumers of UPF was estimated with cross-classification; that is, whether the CUPS scores and the reference method data placed observations in the same quintiles of the CUPS scores and UPF intake. We compared agreement in categorising individuals as low or high consumers based on CUPS scores and the estimated energy from UPF as a reference indicator using the Kappa statistic. We used average values from the CUPS and the corresponding 24HR categorised into quintiles. We used quadratic weighted Kappa, considering that variables were ordinal (quintiles) and to penalise more heavily a larger disagreement^(30)^.

#### Reliability assessment

First, the internal consistency of each CUPS version was examined by calculating inter-item correlations of UPF scores from each screen using Cronbach’s *α*. Second, Pearson’s correlations were used to determine which sub-scores had the greatest influence on the overall score. Third, we assessed the screeners’ reproducibility by calculating the intraclass correlation between scores obtained using the 1-d CUPS 2 weeks apart at T1 and T2. Fourth, to verify the unidimensionality of the CUPS, we conducted a factor analysis on the 30-d CUPS using the twenty-eight sub-categories. Finally, to estimate the extent to which the CUPS produces consistent data across multiple time points, Spearman’s correlations were used to compare the 1-d CUPS score at T1 with the 1-d CUPS score at T2 and the 7-d CUPS with the 30-d CUPS. Spearman’s correlations were also performed between the 7-d CUPS score and the 1-d CUPS scores at T1 and T2, as well as between the 30-d CUPS score and the 1-d CUPS scores at T1 and T2.

#### Associations between Canadian Ultra-Processed Product Screener scores and critical nutrient intake

Spearman’s correlation was used to assess the association between each of the four CUPS scores and intake of critical nutrients (added sugars, Na and saturated fats); high intake of these nutrients has been shown to increase the risk of non-communicable diseases^(31)^. We compared the proportion of individuals in each quintile of the 30-d CUPS scores who exceeded the WHO-recommended intake for free sugars (10 % of energy)^(32)^ and saturated fat (10 % of energy)^(33)^, as well as the Canadian recommendations for Na (2300 mg)^(34,35)^.

#### Reporting and interpretation of results

*P* values were presented for all statistical analyses, and the strength of evidence was interpreted on a scale from no evidence (*P* = 1·0), weak (*P* = 1·0–0·1), moderate (*P* = 0·05–0·01), strong (*P* = 0·01–0·001), to very strong evidence (*P* < 0·001)^(36)^. Correlations were interpreted following Shober *et al.* guidance, with 0–0·10 considered as negligible correlation, 0·10–0·39 as weak, 0·40–0·69 as moderate, 0·70–0·89 as strong and 0·90–1·00 as very strong^(37)^. For the level of agreement between the CUPS score and the reference measurement method (24HR), we followed guidance offered by McHugh to interpret the Kappa statistic (no agreement from 0 to 0·20, fair from 0·21 to 0·39, moderate from 0·40 to 0·59, substantial from 0·60 to 0·79, strong from 0·80 to 0·90 and almost perfect agreement above 0·90)^(38)^. For internal consistency, a Cronbach’s *α* between 0·70 and 0·90 is generally considered an acceptable value^(39)^.

## Results

### Participant characteristics and ultra-processed food intake

Among the 354 participants, 57 % identified their sex at birth as female and 56 % were aged 40 years or older (Table 3). About three-quarters (77 %) had an education above high school level and were English speakers. Participants primarily resided in Ontario (21 %) and Quebec (20 %). The sociodemographic profile of participants who started but did not complete the study (online Supplementary File 1, Table 1) was similar in terms of sex and age, with slightly more individuals with some post-secondary education and living in Ontario.

On average, for the three 24HR, 48·7 % of the daily energy consumed by the participants came from UPF (Table 3). There was no evidence that the mean energy contribution from UPF (averaged from the three recalls) differed by sex, age, education and language, but there was strong evidence for differences by province of residence (*P* = 0·006). The energy contribution from UPF was higher from west to east of the country, with strong evidence that intake was lower in British Columbia (43 %) compared to the Atlantic provinces (55 %) (*P* = 0·004 from a Bonferroni *post hoc* test).

### Canadian Ultra-Processed Product Screener scores and completion time

Table 4 presents the mean and median scores, as well as the minimum and maximum scores for the four CUPS completed by participants. The wide distribution of scores supports the tool’s capacity to distinguish between low and high UPF consumers. For example, the mean score of the 1-d CUPS at T2 was 5/28 and ranged from 0 to 22, while the mean score of the 30-d CUPS was 28·6/140 and ranged from 0 to 99. We found no evidence that the CUPS scores (1-d CUPS at T1 and T2, 7-d and 30-d CUPS) varied according to sex, age, education or language (data not shown). For the 30-d CUPS only, there was weak evidence that the mean scores differed across provinces of residence (*P* = 0·070), with the lowest mean score observed in British Columbia (25·8) and the highest in the Atlantic provinces (33·6) (*P* = 0·069) (Table 3). Table 4 also provides the mean, sd, median, minimum and maximum completion times for the CUPS. Participants with extreme values (who completed the CUPS in more than 1 h) were excluded from the measures presented in Table 4. Mean completion times ranged from 4 min 40 s for the 1-d CUPS at T2 to 6 min 22 s for the 30-d CUPS.

**Table 4.** Descriptive characteristics for the 1-d, 7-d and 30-d CUPS score

	CUPS score					Completion time*				
	Mean	sd	Min	Median	Max	Mean in minutes	sd	Min	Median	Max
CUPS (possible score range)										
1-d CUPS at T1 (0–28)	5·5	3·5	0	5	25	05:45	05:50	01:17	04:28	56:56
1-d CUPS at T2 (0–28)	5·0	3·1	0	4	22	04:40	03:45	01:27	03:39	43:49
7-d CUPS (0–112)	18·2	8·8	0	18	71	05:10	03:33	01:25	04:10	29:39
30-d CUPS (0–140)	28·6	13·7	0	27	99	06:22	05:30	01:43	04:53	45:52

CUPS, Canadian Ultra-Processed Product Screener.

* Number of participants excluded due to extreme values (completed in more than 1 h): 1-d CUPS at T1: *n* 4/1-d CUPS at T2: *n* 4/7-d CUPS: *n* 5/30-d CUPS: *n* 4.

### Construct validity

Strong evidence of moderate correlations was observed between the CUPS scores and the reference measure (i.e. total energy (kcal) from UPF and proportion of energy (% total kcal) from UPF estimated from corresponding 24HR), ranging from 0·33 to 0·44 (*P* < 0·001) (Table 5). Correlations were slightly stronger for longer periods of measurement (i.e. stronger for the 7-d and the 30-d CUPS, compared to the 1-d CUPS at T1 and T2).

**Table 5.** Spearman’s correlation coefficients (*r*) between 1-d, 7-d and 30-d CUPS scores with energy intake from ultra-processed food and (UPF) drink products from the corresponding 24-h recalls

CUPS (correlated with corresponding 24HR)	Total kcal from UPF	*P* value	% of total kcal from UPF	*P* value
1 d (T1)	0·40	< 0·001	0·33	< 0·001
1 d (T2)	0·42	< 0·001	0·35	< 0·001
Mean score of the 1 d at T1 and T2 (mean of T1 + T2)	0·42	< 0·001	0·37	< 0·001
7 d (mean of T1 + T2)	0·44	< 0·001	0·36	< 0·001
30 d (mean of T0 + T1 + T2)	0·44	< 0·001	0·40	< 0·001

CUPS, Canadian Ultra-Processed Product Screener.

Pearson’s correlation analyses were next conducted to assess whether the CUPS scores correlated with their corresponding reference measure and performed better among certain sociodemographic groups (online Supplementary File 1, Table 2). We found that such correlations were stronger for older individuals (30–59 years), individuals with lower educational attainment, English-speaking individuals and residents of Ontario. Correlations for proportion of energy (% kcal) from UPF were slightly lower than the correlations for total energy from UPF for most sociodemographic groups. There were some exceptions where correlation coefficients for proportion of energy from UPF were higher than for total energy from UPF (e.g. French-speaking individuals (*r* = 0·51, *P* < 0·001) and residents of Quebec (*r* = 0·45, *P* < 0·001)).

### Agreement between the Canadian Ultra-Processed Product Screener score and 24HR

The distribution of participants classified into quintiles from low (quintile 1) to high consumers (quintile 5) based on their total energy intake (kcal) from UPF (averaged across three 24HR) and the CUPS scores has fair, but close to moderate agreement for both the 1-d CUPS at T1 (Kappa of 0·39) and T2 (Kappa of 0·39). Moderate agreement was observed for the 7-d CUPS (Kappa of 0·41) and the 30-d CUPS (Kappa of 0·41) (results for the 30-d CUPS are shown in Table 6). Agreement was slightly lower for the proportion of energy (% kcal) from UPF (Kappa of 0·29 for the 1-d CUPS at T1, 0·39 for the 1-d CUPS at T2, 0·35 for the 7-d CUPS and 0·37 for the 30-d CUPS). For the 30-d CUPS, 31 % of individuals were perfectly categorised using the two methods (% UPF or total kcal from UPF), and 68 % of individuals were either perfectly categorised (within the same quintile) or nearly perfectly categorised (within one quintile).

**Table 6.** Distribution of study participants according to quintiles of total energy from ultra-processed food (UPF) and drink products and 30-d CUPS score

Quintile of total energy intake from UPF (kcal)*	Quintiles of the 30-d CUPS score					Total
	Q1-lowest (< 17)	Q2 (18–24)	Q3 (25–31)	Q4 (32–38)	Q5-highest (> 38)	
Q1-lowest (≤ 1648·3)	34	19	8	7	3	70
Q2 (1648·3–2258·8)	16	15	16	13	10	71
Q3 (2258·8–3007·8)	11	16	19	13	12	71
Q4 (3007·8–3956·3)	8	16	20	12	16	72
Q5-highest (≥ 3956·3)	3	10	12	16	29	70
Total	72	76	75	61	70	354

CUPS, Canadian Ultra-Processed Product Screener.

Kappa index: 0·41.

* Total energy (kcal) measured from UPF and drink products averaged across three 24HR.

### Reliability

All versions of the CUPS had a relatively high internal consistency, ranging from 0·63 to 0·86 (Table 2). Internal consistency was higher for the 30-d CUPS (0·86) and the 7-d CUPS (0·80) compared to the 1-d CUPS (0·63 and 0·72).

Next, we assessed how UPF sub-categories’ scores influenced the total scores of the 1-d CUPS at T1 and T2 and the 7-d and 30-d CUPS (online Supplementary File 1, Table 3).

Overall, UPF sub-category scores consistently showed low to moderate correlation with the total score from their respective screener. For instance, for the 1-d CUPS at T1, all sub-categories showed low to moderate levels of correlation with the total score, ranging from 0·21 (*P* < 0·001) for protein powder to 0·43 (*P* < 0·001) for fruit and vegetable juices and drinks or iced tea. Lower correlations were found between UPF sub-category scores and the percentage of kcal from UPF compared to correlations with the total score.

To verify the unidimensionality of the CUPS, we conducted a factor analysis on the 30-d CUPS using the twenty-eight-item sub-categories. We obtained a six-factor solution, with a dominant first factor explaining 22 % of the variance (eigenvalues of 5·9). The other factors explained between 4 % and 6 % of the total variance (eigenvalues of 1·3 to 1·7). The second factor included three items with high loadings: plant-based drinks, plant-based meats and protein powders. Similar results were obtained with factor analyses of the 1-d and 7-d CUPS, with the dominant first factor explaining 11 % and 14 % of the variance, respectively (data not shown).

We found strong evidence that the CUPS was reproducible over time (2 weeks) with an intraclass correlation of 0·62 (0·55 to 0·68) and an *F* test of 4·20 (< 0·001). Spearman’s correlation coefficients were 0·64 (*P* < 0·001) between the 1-d CUPS score at T1 and 1-d CUPS score at T2, and 0·83 (< 0·001) between the 30-d and 7-d CUPS scores. Coefficient correlations were 0·73 and 0·63 (*P* < 0·001) between the 7-d CUPS score and the 1-d CUPS scores at T1 and T2, respectively, and 0·67 and 0·65 (*P* < 0·001) between the 30-d CUPS score and the 1-d CUPS scores at T1 and T2, respectively.

#### Associations with critical nutrients

Table 7 presents results from Spearman’s correlation analysis between intakes of critical nutrients (i.e. added sugars, Na and saturated fats) and CUPS scores. The CUPS mean scores were weakly to moderately correlated with energy (kcal) and relative energy (% kcal) from added sugars and saturated fats, as well as Na intake (mg). The CUPS mean scores showed weaker correlations than when intakes from these nutrients are compared with the reference method (% kcal reported in the 24HR at T0-T1-T2).

**Table 7.** Spearman’s correlation coefficient (*r*) between mean CUPS scores and intakes of added sugars, saturated fats and sodium

	1-d CUPS at T1		1-d CUPS at T2		7-d CUPS		30-d CUPS		% kcal from UPF based on 24HR at T0, T1, T2	
	*r*	*P* value	*r*	*P* value	*r*	*P* value	*r*	*P* value	*r*	*P* value
Added sugars (kcal)	0·29	< 0·001	0·40	< 0·001	0·32	< 0·001	0·38	< 0·001	0·51	< 0·001
Added sugars (% kcal)	0·21	< 0·001	0·35	< 0·001	0·24	< 0·001	0·31	< 0·001	0·54	< 0·001
Na (mg)	0·24	< 0·001	0·25	< 0·001	0·24	< 0·001	0·27	< 0·001	0·16	0·003
Saturated fats (kcal)	0·24	< 0·001	0·28	< 0·001	0·19	< 0·001	0·31	< 0·001	0·21	< 0·001
Saturated fats (% kcal)	0·04	0·477	0·11	0·038	0·07	0·901	0·12	0·019	0·11	0·047

CUPS, Canadian Ultra-Processed Product Screener; UPF, ultra-processed food.

To illustrate the association between the CUPS score and intake of critical nutrients, we compared individuals in the highest *v*. the lowest quintile of the 30-d CUPS score (Table 8). Compared with participants in the lowest CUPS score quintile, participants in the highest quintile consumed nearly 1000 mg more Na per d (2836 mg in Q1 *v*. 3729 mg in Q5), twice as much energy from added sugars (Q1: 446 *v*. Q5: 906 kcal) and 210 more kcal from saturated fats (Q1: 571 *v*. Q5: 781 kcal).

**Table 8.** Quintile distribution of the 30-d CUPS scores and intakes of critical nutrients estimated using three 24HR

Quintiles of 30-d CUPS score (mean; min-max)	Total daily energy intake (kcal)^*^	Added sugars (kcal per d)	Added sugars (% kcal)	Saturated fats (kcal per d)	Saturated fats (%kcal)	Sodium (mg/d)
Q1 (11·9; 0–17)	1624·2	446·0	7·8	570·5	11·6	2835·9
Q2 (21·2; 18–24)	1869·4	569·1	8·6	714·8	12·4	3212·3
Q3 (28·0; 25–31)	1915·0	628·1	9·6	701·5	12·2	3358·1
Q4 (34·7; 32–38)	1931·9	840·8	12·2	730·4	12·5	3419·0
Q5 (49·2; 39–99)	2150·7	905·8	12·1	780·6	12·1	3729·2

CUPS, Canadian Ultra-Processed Product Screener.

* Total energy (kcal) measured from ultra-processed food (UPF) and drink products averaged across three 24HR.

**Figure 3.**
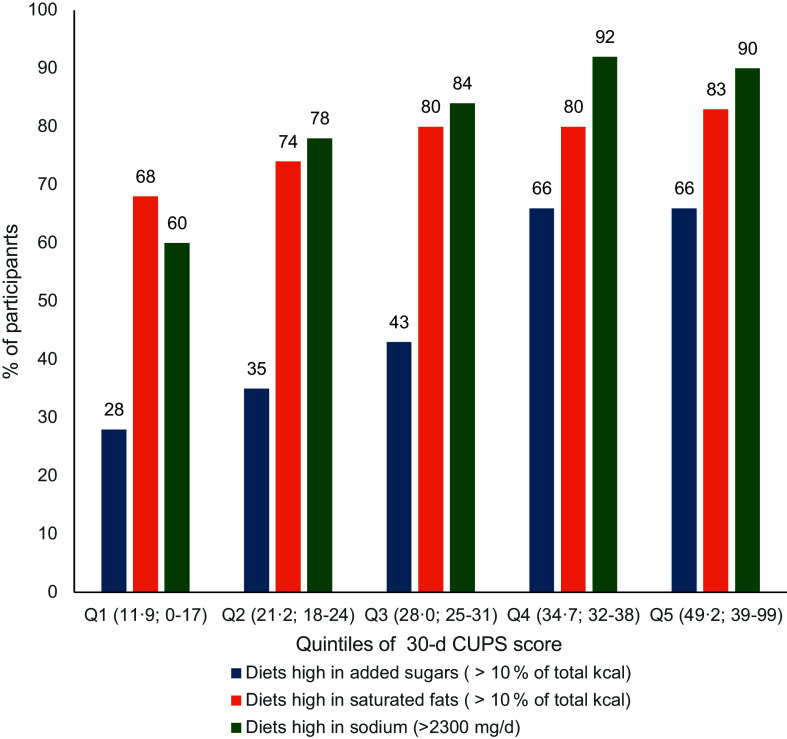
Proportion of participants with diets high in added sugars, saturated fats and sodium assessed by 24-h recalls at T0, T1 and T2 according to quintiles of the 30-d CUPS score. Q# (X ; X-X) = Quintile# (Mean score; Minimum-Maximum score). CUPS, Canadian Ultra-Processed Product Screener.

Globally, we observed that intake of critical nutrients increased along with increasing intake of UPF measured by the 30-d CUPS score, as shown in Figure 3. For instance, about a quarter (28 %) of individuals in the lowest quintile (Q1) of the 30-d CUPS score had diets high in added sugars (i.e. > 10 % kcal from added sugars) compared with 66 % of individuals in quintiles 4 and 5. It should be noted that the WHO recommendations include both added sugars and free sugars, which means that these results are likely conservative.

## Discussion

This study suggests that the CUPS has acceptable validity and reliability for its intended use in the Canadian context, which is to quickly distinguish between high and low consumers of UPF among adults. The CUPS has an acceptable construct validity for a screener, with moderate correlations between the CUPS score and the consumption level of UPF (for total energy from UPF and proportion of energy from UPF) measured using multiple 24HR. Moderate agreement was also found between categorising individuals in quintiles from low to high consumers based on their CUPS scores and the estimated consumption of total energy from UPF and proportion of energy from UPF as a reference indicator.

Differences in UPF intake across provinces of residence were found when using the reference measure (24HR), especially between individuals from the Atlantic provinces and British Columbia. Similar but weaker evidence of differences was observed with the CUPS, mainly at 7-d and 30-d, possibly due to a lack of statistical power, given that only forty individuals were from the Atlantic provinces. There was little to no evidence of differences in CUPS scores according to other sociodemographic variables, and the findings were similar when using the 24HR measure. These results are unsurprising, considering UPF intake in Canada is high across all sociodemographic groups, with minor differences by sex, income and educational attainment^(4,40)^. This study’s failure to observe evidence of differences across these groups could be explained by our sample size and its sociodemographic distribution, as our sample was more educated than the general population and included few younger adults (18–21 years).

The CUPS had an acceptable level of reproducibility with a moderately strong intraclass correlation and a very good internal consistency. As shown, the 30-d CUPS was the version that performed best in all validity and reliability assessments and may be more appropriate to gather more precise and reliable data over a longer time frame. Our factor analyses indicate that the CUPS lean towards unidimensionality while suggesting the existence of specific patterns of UPF intake, such as individuals consuming plant-based alternatives or UPF perceived as ‘healthy’ (e.g. protein powders). The internal consistency was also higher with a more extended reference period (7-d or 30-d CUPS).

The level of correlation between the scores and the reference measure observed in this study aligns with other screeners. For example, the Behavioral Risk Factor Surveillance System in the USA, which includes a six-item brief dietary assessment of fruit and vegetable intake, has correlation coefficients ranging from 0·29 to 0·54 when compared with multiple diet records and 24HR^(41)^. Overall, the reliability assessment of the CUPS is also comparable to other tools that measure diet quality in Canada, namely, the Healthy Eating Food Index (HEFI) 2019 and the Canadian Food Intake Screener, which assess the alignment of adults’ dietary intakes with the 2019 Canada Food Guide’s healthy food choices^(7,42)^. At present, the CUPS internal consistency is similar to or higher than the HEFI 2019 (Cronbach *α* of 0·63 to 0·80 compared to 0·66). This is not surprising considering that UPF is thought of as a unidimensional construct that can be used as an overall measure of one key dimension of diet quality, while the HEFI is a multicomponent measure^(42)^.

Based on our results, the CUPS allows to identify diets higher in added sugars, saturated fats and Na, as well as individuals who exceed the recommended limits^(32–34)^ of these critical nutrients associated with obesity and non-communicable diseases^(31)^. However, our results also indicate the need to complement the CUPS with assessments of culinary ingredient use (such as salt and sugar) and the intake of plant- *v*. animal-based products to identify diets that are low in UPF but high in essential nutrients, considering salt use in meals preparation with fresh and minimally processed foods and consumption of non-UPF high in saturated fats like meats and dairy products.

Another screener estimating UPF intake, the NOVA score, was developed in Brazil and adapted in countries such as Senegal and Ecuador^(43)^. The NOVA score was identified and assessed by the Healthy Diets Monitoring Initiative (HDMI) as a valid, useful and fit-for-purpose healthy diet metric to use as a national indicator^(43)^. In Brazil, Costa *et al.* found substantial agreement (Pabak index of 0·67) between the UPF score and the estimated consumption level of UPF using assisted 24HR recalls^(44)^. In Senegal, Kébé *et al.* also found a high agreement between their UPF score and the assisted 24HR (Pabak index of 0·84)^(45)^. Neither study reported an assessment of internal consistency. In addition, our results cannot be directly compared to these assessments because the CUPS was not administered using dietitian assistance as in the Kébé *et al.* and Costa *et al.* studies, meaning that 24HR completed by participants in the current study may be of lower precision. In Canada, as in other high-income countries, there is also more diversity of processed foods and UPF in the market, thus making the distinction between non-UPF and UPF less obvious for consumers compared with Senegal and Brazil, where UPF markets are still emerging (e.g. in Senegal, 17 % of kcal consumed comes from UPF)^(45,46)^.

The CUPS can be self-administered in about 2–4 min (1-d CUPS) and 5–6 min (7-d or 30-d CUPS) and was designed to be simple and easy to understand. Given that level of UPF consumption has been recognised as an indicator of diet quality, the CUPS score can be used in surveillance, cohort studies and interventions aimed at the prevention and reversal of obesity and other diet-related conditions in Canada^(43,47)^. Finally, this study contributes to global efforts to develop tools for assessing UPF intake, alongside countries such as Brazil, Senegal, India and Ecuador^(43)^.

### Strengths and limitations

First, we conducted several validity and reliability assessment tests that provide a complete overview of the validity of the screener for its use and purpose. We were also able to compare the psychometric properties of the CUPS over different periods of time and in both official languages of Canada. Our sample included Canadian adults from all provinces and all age groups. However, our sample is skewed towards higher education and thus potentially higher food literacy. Our results suggest that the CUPS performs better among people with lower levels of education compared with higher education, suggesting potential differences in the identification or health perceptions of UPF (some UPF are targeted to higher literacy consumers and may be perceived as healthy and not ultra-processed).

An important limitation that affected our results is that 24HR were completed by participants without assistance by a trained dietitian, contrary to Kébé *et al.* and Costa *et al.* studies. This may have created more recall bias^(48)^. Survey fatigue due to having numerous questionnaires over a long period of time may have also affected motivation and responses, which may explain the high level of attrition over the study period and could also increase the likelihood of sample bias (more motivated or educated participants completed the study). Future research should aim to further evaluate the CUPS in more socially and culturally diverse populations and use different reference methods of dietary assessment to improve the quality of data collected.

### Conclusion

The CUPS has an acceptable construct validity and level of reproducibility to estimate UPF consumption among adults in Canada. The score is useful for identifying low and high consumers of these products and dietary patterns higher in critical nutrients (added sugars, saturated fats and Na). The CUPS could serve as a proxy measure for one key dimension of diet quality, which is the type of food processing, and contribute to global efforts to develop tools for assessing UPF intake.

## Supporting information

Hamel et al. supplementary material Hamel et al. supplementary material

## Supplementary material

For supplementary material accompanying this paper visit https://doi.org/10.1017/S1368980026102304
