# Supplementary material for: Assessment of construct validity and reliability of the Canadian Ultra-Processed Product Screener
Source: Public Health Nutr. 2026 Mar 9;29(1):e69. doi: 10.1017/S1368980026102304 (PMC13087981; doi:10.1017/S1368980026102304)
Supplement: Hamel et al. supplementary material [file S1368980026102304sup001.pdf]

Supplementary file 1, Supplementary tables 1,2,3

Table 1. Sociodemographic characteristics of participants who started the study but did not complete it, n = 314 (completed at least one questionnaire)

	Distribution of participants % (n)
Sex at birth	
Male	46.2 (145)
Female	53.8 (169)
Age (years)	
18-29	16.6 (52)
30-39	27.4 (86)
40-49	24.8 (78)
50-59	31.2 (98)
Educational attainment*	
High school or less	22.6 (80)
Some postsecondary	26.8 (95)
University degree or above	50.6 (179)
Language	
English	79.9 (251)
French	20.1 (63)
Province†	
Atlantic provinces	11.1 (35)
Quebec	15.9 (50)
Ontario	29.3 (92)
Prairie provinces	25.5 (80)
British Columbia	18.2 (57)

*Postsecondary includes trade certificate or diploma, college, CEGEP (College of general and professional education unique to Quebec province) or other non-university certificate or diploma, and university certificate or diploma below bachelor level. One participant refused to answer.

†Atlantic provinces include New Brunswick, Prince Edward Island, Nova Scotia, and Newfoundland and Labrador. Prairie provinces include Alberta, Saskatchewan, and Manitoba.

Table 2. Pearson correlation coefficient (r) between 1-day, 7-day, and 30-day CUPS scores with energy intake (kcal) from UPF and proportion of total energy (% kcal) from Ultra-processed food and drink products (UPF) estimated from the corresponding 24-HR, according to sociodemographic characteristics (n=354)

	**1-day CUPS and 24HR at T1, r (p-value)**		**1-day CUPS and 24HR at T2, r (p-value)**		**7-day CUPS and 24HR at T0-T1, r (p-value)**		**30-day CUPS and 24HR at T0-T1-T2, r (p-value)**	
	UPF kcal	% UPF Kcal	UPF kcal	% UPF Kcal	UPF kcal	% UPF Kcal	UPF kcal	% UPF Kcal
**Sex**								
Male (n=154)	0.402 (<0.001)	0.319 (<0.001)	0.294 (<0.001)	0.304 (<0.001)	0.482 (<0.001)	0.428 (<0.001)	0.492 (<0.001)	0.416 (<0.001)
Female (n=200)	0.238 (<0.001)	0.184 (0.032)	0.469 (<0.001)	0.201 (0.004)	0.448 (<0.001)	0.323 (<0.001)	0.479 (<0.001)	0.391 (<0.001)
**Age (years)**								
18-29 (n=77)	0.096 (0.407)	1.171 (0.136)	0.448 (<0.001)	0.246 (0.031)	0.325 (0.004)	0.267 (0.019)	0.334 (0.003)	0.203 (0.077)
30-39 (n=78)	0.257 (0.023)	0.353 (0.002)	0.317 (0.005)	0.130 (0.257)	0.493 (<0.001)	0.448 (<0.001)	0.538 (<0.001)	0.496 (<0.001)
40-49 (n=102)	0.394 (<0.001)	0.203 (0.041)	0.290 (0.003)	0.341 (<0.001)	0.400 (<0.001)	0.338 (<0.001)	0.439 (<0.001)	0.453 (<0.001)
50-59 (n=97)		0.277 (0.006)	0.535 (<0.001)	0.260 (0.010)	0.590 (<0.001)	0.425 (<0.001)	0.576 (<0.001)	0.451 (<0.001)
**Educational attainment**								
High school or less (n=80)	0.502 (<0.001)	0.136 (0.229)	0.615 (<0.001)	0.301 (0.007)	0.512 (<0.001)	0.378 (<0.001)	0.663 (<0.001)	0.488 (<0.001)
Some postsecondary* (n=95)	0.156 (0.132)	0.272 (0.008)	0.463 (<0.001)	0.256 (<0.012)	0.472 (<0.001)	0.387 (<0.001)	0.505 (<0.001)	0.429 (<0.001)
University degree or above (n=179)	0.226 (0.002)	0.277 (<0.001)	0.251 (<0.001)	0.238 (0.001)	0.430 (<0.001)	0.366 (<0.001)	0.371 (<0.001)	0.343 (<0.001)
**Language**								
English (n=274)	0.315 (<0.001)	0.205 (<0.001)	0.428 (<0.001)	0.280 (<0.001)	0.474 (<0.001)	0.333 (<0.001)	0.506 (<0.001)	0.369 (<0.001)
French (n=80)	0.256 (0.022)	0.430 (<0.001)	0.251 (0.025)	0.177 (0.116)	0.422 (<0.001)	0.528 (<0.001)	0.400 (<0.001)	0.507 (<0.001)
**Province**								
Atlantic provinces (n=40)	0.598 (<0.001)	0.515 (<0.001)	0.407 (0.009)	0.083 (0.610)	0.540 (<0.001)	0.425 (0.006)	0.583 (<0.001)	0.256 (0.111)
Quebec (n=72)	0.234 (0.048)	0.324 (0.005)	0.174 (0.145)	0.132 (0.269)	0.382 (<0.001)	0.480 (<0.001)	0.355 (0.02)	0.454 (<0.001)
Ontario (n=74)	0.454 (<0.001)	0.198 (0.092)	0.573 (<0.001)	0.448 (<0.001)	0.552 (<0.001)	0.410 (<0.001)	0.640 (<0.001)	0.501 (<0.001)
Prairie province (n=100)	0.078 (0.440)	0.132 (0.189)	0.417 (<0.001)	0.192 (0.055)	0.425 (<0.001)	0.266 (0.008)	0.397 (<0.001)	0.266 (0.007)
British Columbia (n=68)	0.315 (0.009)	0.302 (0.012)	0.350 (0.003)	0.316 (0.009)	0.386 (0.001)	0.311 (0.010)	0.416 (<0.001)	0.410 (<0.001)

Table 3. Pearson correlation coefficients (r) between ultra-processed food and drink products sub-category scores with total scores from the 1-day, 7-day, and 30-day CUPS and between CUPS sub-category scores and proportion of total energy (% kcal) from UPF estimated from the corresponding 24HR

UPF sub-category	1-day CUPS and 24HR at T1, r (p-value)		1-day CUPS and 24HR at T2, r (p-value)		7-day CUPS and 24HR at T0-T1, r (p-value)		30-day CUPS and 24HR at T0-T1-T2, r (p-value)	
	UPF sub-category score and total score	UPF sub-category score and % UPF Kcal at T1	UPF sub-category score and total score	UPF sub-category score and % UPF Kcal at T2	UPF sub-category score and total score	UPF sub-category score and UPF Kcal at T0-T1	UPF sub-category score and total score	UPF sub-category score and % UPF Kcal at T0-T1-T2
**Fruit and vegetable juices and drinks or iced tea**	0.431 (<0.001)	0.026 (0.627)	0.463 (<0.001)	0.069 (0.194)	0.430 (<0.001)	0.137 (0.010)	0.476 (<0.001)	0.239 (<0.001)
**Coffee or tea drinks**	0.305 (<0.001)	0.056 (0.295)	0.423 (<0.001)	0.120 (0.024)	0.353 (<0.001)	0.043 (0.424)	0.414 (<0.001)	0.153 (0.004)
**Sports or energy drinks**	0.417 (<0.001)	0.072 (0.175)	0.381 (<0.001)	0.154 (0.004)	0.389 (<0.001)	0.037 (0.489)	0.407 (<0.001)	0.116 (0.029)
**Soft drinks or flavoured sparkling water**	0.343 (<0.001)	0.140 (0.009)	0.323 (<0.001)	0.193 (<0.001)	0.424 (<0.001)	0.292 (<0.001)	0.443 (<0.001)	0.278 (<0.001)
**Chocolate milk**	0.264 (<0.001)	-0.025 (0.642)	0.072 (0.177)	-0.077 (0.146)	0.260 (<0.001)	0.050 (0.348)	0.310 (<0.001)	0.064 (0.228)
**Plant-based drinks**	0.211 (<0.001)	0.034 (0.521)	0.171 (<0.001)	0.024 (0.654)	0.121 (0.023)	-0.050 (0.349)	0.108 (0.042)	-0.60 (0.256)
**Breakfast cereals**	0.371 (<0.001)	0.047 (0.378)	0.230 (<0.001)	0.044 (0.412)	0.318 (<0.001)	0.090 (0.093)	0.487 (<0.001)	0.203 (<0.001)
**Commercial baked goods**	0.380 (<0.001)	0.114 (0.031)	0.327 (<0.001)	0.050 (0.345)	0.377 (<0.001)	0.219 (<0.001)	0.529 (<0.001)	0.278 (<0.001)
**Fast-food products**	0.277 (<0.001)	0.037 (0.483)	0.256 (<0.001)	0.113 (0.034)	0.369 (<0.001)	0.281 (<0.001)	0.535 (<0.001)	0.325 (<0.001)
**Commercial breads**	0.389 (<0.001)	0.160 (0.002)	0.290 (<0.001)	0.078 (0.141)	0.449 (<.001)	0.299 (<0.001)	0.542 (<0.001)	0.252 (<0.001)
**Processed meat or seafood**	0.400 (<0.001)	0.065 (0.221)	0.322 (<0.001)	0.064 (0.233)	0.471 (<0.001)	0.153 (0.004)	0.592 (<0.001)	0.233 (<0.001)
**Plant-based meat and seafood alternatives**	0.335 (<0.001)	0.011 (0.832)	0.351 (<0.001)	0.092 (0.083)	0.131 (<0.001)	0.065 (0.224)	0.294 (<0.001)	0.127 (0.017)
**Cheese products**	0.410 (<0.001)	0.136 (0.010)	0.414 (<0.001)	0.023 (0.671)	0.424 (<0.001)	0.148 (0.005)	0.539 (<0.001)	0.247 (<0.001)
**Instant, canned, or packaged soups and noodles**	0.395 (<0.001)	0.106 (0.046)	0.297 (<0.001)	0.032 (0.545)	0.360 (<0.001)	0.205 (<0.001)	0.520 (<0.001)	0.225 (<0.001)
**Frozen or fast-food pizzas**	0.307 (<0.001)	0.037 (0.487)	0.176 (<0.001)	0.003 (0.958)	0.377 (<0.001)	0.228 (<0.001)	0.452 (<0.001)	0.215 (<0.001)
**Ready-to-cook or frozen meals**	0.273 (<0.001)	0.160 (0.003)	0.294 (<0.001)	0.106 (0.047)	0.416 (<0.001)	0.098 (0.065)	0.510 (<0.001)	0.286 (<0.001)
**Snack bars**	0.323 (<0.001)	0.086 (0.107)	0.237 (<0.001)	0.073 (0.168)	0.327 (<0.001)	0.197 (<0.001)	0.417 (<0.001)	0.228 (<0.001)
**Flavoured yogurts or puddings**	0.248 (<0.001)	0.068 (0.205)	0.343 (<0.001)	0.026 (0.631)	0.354 (<0.001)	0.074 (0.164)	0.442 (<0.001)	0.072 (0.172)
**Chips, crackers, or other salthy snacks**	0.311 (<0.001)	0.071 (0.185)	0.348 (<0.001)	0.128 (0.016)	0.416 (<0.001)	0.174 (<0.001)	0.518 (<0.001)	0.227 (<0.001)
**Chocolates or candies**	0.315 (<0.001)	0.118 (0.027)	0.303 (<0.001)	0.156 (0.003)	0.398 (<0.001)	0.275 (<0.001)	0.491 (<0.001)	0.258 (<0.001)
**Ice cream, popsicles, or frozen desserts**	0.387 (<0.001)	0.068 (0.205)	0.291 (<0.001)	0.082 (0.122)	0.344 (<0.001)	0.136 (0.011)	0.458 (<0.001)	0.134 (0.016)
**Spreads or jams**	0.419 (<0.001)	0.137 (0.010)	0.377 (<0.001)	0.130 (0.014)	0.435 (<0.001)	0.153 (0.004)	0.445 (<0.001)	0.177 (<0.001)
**Margarine or shortening**	0.282 (<0.001)	0.226 (<0.001)	0.362 (<0.001)	0.091 (0.087)	0.398 (<0.001)	0.181 (<0.001)	0.449 (<0.001)	0.223 (<0.001)
**Sauces or condiments**	0.336 (<0.001)	0.070 (0.190)	0.359 (<0.001)	0.071 (0.183)	0.468 (<0.001)	0.060 (0.262)	0.550 (<0.001)	0.216 (<0.001)
**Salad dressings**	0.281 (<0.001)	0.067 (0.208)	0.164 (0.002)	-0.055 (0.305)	0.279 (<0.001)	0.039 (0.469)	0.487 (<0.001)	0.176 (0.001)

**Broths or seasonings**	0.227 (<0.001)	0.010 (0.847)	0.299 (<0.001)	-0.007 (0.895)	0.295 (<0.001)	0.051 (0.341)	0.373 (<0.001)	0.068 (0.199)
**Sugar substitutes or coffee whiteners**	0.360 (<0.001)	0.079 (0.139)	0.317 (<0.001)	0.111 (0.038)	0.321 (<0.001)	0.080 (0.135)	0.365 (<0.001)	0.139 (0.009)
**Protein powder or meal replacements**	0.208 (<0.001)	-0.118 (0.027)	0.177 (<0.001)	-0.003 (0.953)	0.146 (0.006)	-0.150 (0.005)	0.150 (0.005)	-0.165 (0.002)
